# Inhibition of SERT and NMDAR synergistically confers rapid antidepressant effects of ketamine

**DOI:** 10.1093/nsr/nwaf367

**Published:** 2025-09-08

**Authors:** Huoqing Luo, Ming Chen, Yingjie Ning, Li Ren, Yiping Lu, Junyou Sun, Xiaona Zhu, Mingzi Ran, Juan Guo, Chen Lu, Chengyu Fan, Jianjun Cheng, Weimin Zheng, Yue Hu, Tangsheng Lu, Gang Wang, Wenzhi Sun, Hailong Dong, Jingpeng Ge, Ji Hu

**Affiliations:** Department of Anesthesiology and Perioperative Medicine, Xijing Hospital, Fourth Military Medical University, Xi'an 710032, China; School of Life Science and Technology, ShanghaiTech University, Shanghai 201210, China; MOE Frontier Center for Brain Science, Institutes of Brain Science, Fudan University, Shanghai 200032, China; School of Life Science and Technology, ShanghaiTech University, Shanghai 201210, China; Lin Gang Laboratory, Shanghai 200031, China; School of Basic Medical Sciences, Chengdu University of Traditional Chinese Medicine, Chengdu 611137, China; Chinese Institute for Brain Research, Beijing 102206, China; Academy for Advanced Interdisciplinary Studies, Peking University, Beijing 100871, China; Chinese Institute for Brain Research, Beijing 102206, China; School of Basic Medical Sciences, Capital Medical University, Beijing 100069, China; School of Life Science and Technology, ShanghaiTech University, Shanghai 201210, China; Department of Anesthesiology and Perioperative Medicine, Xijing Hospital, Fourth Military Medical University, Xi'an 710032, China; Department of Anesthesiology and Perioperative Medicine, Xijing Hospital, Fourth Military Medical University, Xi'an 710032, China; School of Life Science and Technology, ShanghaiTech University, Shanghai 201210, China; School of Life Science and Technology, ShanghaiTech University, Shanghai 201210, China; iHuman Institute, ShanghaiTech University, Shanghai 201210, China; School of Pharmacy, Nanjing Medical University, Nanjing 211166, China; Department of Anesthesiology, Huashan Hospital, Fudan University, Shanghai 200040, China; National Institute on Drug Dependence and Beijing Key Laboratory of Drug Dependence, Peking University, Beijing 100191, China; Beijing Key Laboratory of Mental Disorders, National Clinical Research Center for Mental Disorders & National Center for Mental Disorders, Beijing Anding Hospital, Capital Medical University, Beijing 100088, China; Chinese Institute for Brain Research, Beijing 102206, China; Department of Anesthesiology and Perioperative Medicine, Xijing Hospital, Fourth Military Medical University, Xi'an 710032, China; School of Life Science and Technology, ShanghaiTech University, Shanghai 201210, China; School of Life Science and Technology, ShanghaiTech University, Shanghai 201210, China; Shanghai Key Laboratory of Psychotic Disorders, Shanghai Mental Health Center, Shanghai 200030, China

**Keywords:** ketamine, rapid antidepressant effects, SERT, NMDAR, vasoactive intestinal peptide-expressing interneurons

## Abstract

While *N*-methyl-d-aspartate receptor (NMDAR) blockade is crucial for the rapid antidepressant effects of ketamine, the involvement of other mechanisms remains contentious, particularly regarding the role of serotonin, a key neurotransmitter in the target of traditional antidepressants. Here, we demonstrate that ketamine elevates serotonin levels by inhibiting the serotonin transporter (SERT). A cryogenic electron microscopy structure of ketamine-bound SERT in the outward-open conformation, resolved at 3.2 Å, indicates that ketamine binds to the central site of SERT. Elevated serotonin, along with NMDAR inhibition, induces ketamine-like rapid antidepressant effects. This increase in serotonin leads to the activation of vasoactive intestinal peptide (VIP)-expressing interneurons, which are essential for the rapid antidepressant effects of ketamine. Inhibition of VIP neurons blocks these effects and ketamine-like effects, highlighting a crucial cell type-specific mechanism. These findings identify a critical pathway in the rapid antidepressant actions of ketamine and offer potential pharmacological strategies for developing rapidly acting antidepressants.

## INTRODUCTION

Major depressive disorder is characterized by a persistently low mood and the loss of interest in activities, resulting in substantial impairment of daily life [1]. Conventional antidepressants, such as selective serotonin (5-HT) reuptake inhibitors (SSRIs), often take weeks to months to achieve their full effects [2–6]. Remarkably, a single sub-anesthetic administration of the *N*-methyl-d-aspartate receptor (NMDAR) antagonist ketamine elicits rapid onset and sustained antidepressant effects, even in treatment-resistant patients [7–14]. Although accumulating evidence indicates that ketamine exerts its rapid antidepressant effects through the inhibition of NMDAR [15–24], several NMDAR inhibitors fail to produce ketamine-like rapid antidepressant effects in clinical trials [24–35].

The serotonergic system is important in emotional regulation and mediating antidepressant effects [36–42]. Inhibiting serotonin transporter (SERT) to increase extracellular 5-HT is considered the primary mechanism of action for traditional antidepressants such as SSRIs [4,43–47]. It is noteworthy that the serotonergic system may also be involved in the rapid antidepressant effects of ketamine [48,49]. Ketamine fails to elicit rapid and sustained antidepressant effects in either SERT knockout mice or mice with 5-HT depletion [25,50]; however, the precise mechanisms by which the serotonergic system contributes to ketamine's antidepressant effects remain elusive.

In this study, using cryogenic electron microscopy (cryo-EM), radioligand binding assays, *in vivo* microdialysis and positron emission

tomography (PET) techniques, we demonstrate that ketamine directly binds to the central pocket of SERT, thereby inhibiting the reuptake of 5-HT by SERT and elevating the concentration of 5-HT in the synaptic cleft. Notably, our study indicates that combining SSRIs with another NMDAR inhibitor, memantine, which does not have antidepressant effects [34], can produce rapid antidepressant effects similar to those of ketamine. Cortical interneurons have been suggested to be very important for ketamine's rapid antidepressant effects [51–53]. Here, our results indicate that ketamine's synergistic inhibition of SERT and NMDAR leads to specific activation of vasoactive intestinal peptide (VIP) neurons in the medial prefrontal cortex (mPFC). Furthermore, we establish that this selective VIP neuronal activation is critical for ketamine's rapid antidepressant effects.

## RESULTS

### Ketamine elevates mPFC 5-HT levels by inhibiting SERT

**Figure 1.**
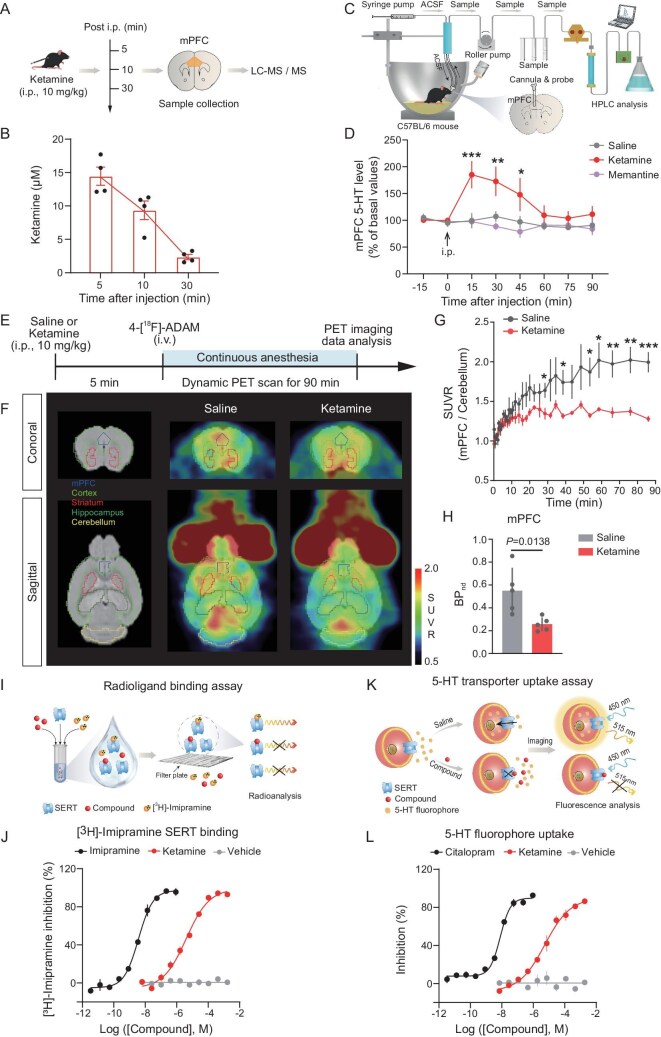
Ketamine inhibits SERT to elevate 5-HT levels. (A) Schematic of LC-MS/MS after intraperitoneal (i.p.) injection of ketamine. (B) Brain concentrations of ketamine after a single i.p. injection of ketamine in mice, as measured by LC-MS/MS (four mice per group). (C) *In vivo* real-time microdialysis setup and experimental procedure. HPLC, high-performance liquid chromatography. (D) Concentration of mPFC 5-HT before and after drug injection (saline: six mice; ketamine: six mice). Ketamine (i.p., 10 mg/kg), Memantine (i.p., 10 mg/kg). Two-way ANOVA with Bonferroni's multiple comparisons test. (E) Workflow of the dynamic PET imaging (0–90 min) study with 4-[^18^F]-ADAM. (F) Representative PET images of a mouse brain after saline and ketamine injection. SUVR, standard uptake value ratio. (G) Quantification of 4-[^18^F]-ADAM SUVR for the mPFC relative to the cerebellum (saline: five mice; ketamine: five mice). Multiple unpaired *t*-tests. (H) BP_nd_ values after treatment with saline or ketamine in the mPFC. Two-tailed unpaired *t*-test. (I) Experimental procedure for the radioligand binding assay. (J) Dose-dependent inhibition of [^3^H]-imipramine radioligand binding to the cell membrane of human cells that express human SERT separated from the cytoplasm by ketamine in the radioligand binding assay. (K) 5-HT transporter uptake experimental procedure. Excitation wavelength: 450 nm; emission wavelength: 515 nm. (L) The dose-dependent inhibition of 5-HT fluorophore binding to SERT by ketamine in the 5-HT transporter uptake experiment. Data are shown as the mean ± SEM. *P < 0.05; **P < 0.01; ***P < 0.001 (see Table S3 for statistical analyses and *n* numbers).

We first used liquid chromatography-tandem mass spectrometry (LC-MS/MS) to detect the concentrations of ketamine in the PFC at different timepoints after intraperitoneal injection (Fig. 1A). mPFC concentrations of ketamine gradually dropped over 30 min after injection (Fig. 1B). To examine whether ketamine increases extracellular levels of 5-HT in freely behaving mice [49], we performed real-time *in vivo* microdialysis to measure the mPFC 5-HT concentration (Fig. 1C). We noted a rapid increase in the average relative-to-baseline 5-HT levels from ∼100% to ∼180% in the mPFC during the 15 min after ketamine injection. Still, memantine did not show such an effect (Fig. 1D). Notably, ketamine did not activate 5-HT neurons in the dorsal raphe nucleus (DRN) (Fig. S1A–F), which indicates that the increase in mPFC 5-HT levels is due to ketamine inhibition of SERT, rather than the activation of DRN 5-HT neurons or inhibition of NMDAR. To further assess ketamine inhibition of SERT in the mouse brain *in vivo*, we performed PET-computed tomography (PET-CT) imaging with *N,N*-dimethyl-2-(2-amino-4-[^18^F] fluorophenylthio) benzylamine (4-[^18^F]-ADAM), a highly selective radioligand for SERT imaging [54]. A dynamic, 90 min PET-CT scan was started after injection of 4-[^18^F]-ADAM (Fig. 1E). PET-CT imaging indicated that ketamine was able to inhibit 4-[^18^F]-ADAM binding to cortical SERT (Fig. 1F). The standard uptake value ratio data showed that ketamine significantly reduces the uptake level of 4-[^18^F]-ADAM in the mPFC (Fig. 1G). Furthermore, the SERT non-displaceable binding potential (BPnd) value of the ketamine group had an average 53.41% decrease compared to the control group (Fig. 1H), indicating that ketamine enhanced serotonergic transmission by inhibition of SERT activity.

To explore the function of SERT, we carried out saturation experiments using high-affinity [^3^H]-imipramine, for which we determined a dissociation constant (*K*_d_) of 2.42 nM (Table S1). To characterize ketamine binding to SERT, we carried out competition experiments (Fig. 1I), with imipramine as the positive control in combination with [^3^H]-imipramine and measured a half-maximal inhibitory concentration (*IC*_50_) of 4.49 μM for ketamine and 3.17 nM for imipramine (Fig. 1J), and an inhibition constant (*K*_i_) of 2.45 μM for ketamine and 1.73 nM for imipramine (Table S1). Thus, both ketamine and imipramine compete for [^3^H]-imipramine binding.

To further assess whether ketamine affects the reuptake of 5-HT through the SERT, we performed a homogeneous assay for 5-HT transporter activity based on cellular uptake of a fluorescent dye coupled with a proprietary masking dye (Fig. 1K). We determined the Michaelis–Menten constant (*K*_m_) for the fluorescent substrate mimetic for SERT to be 0.96 μM (Table S1), with citalopram as a positive control. Ketamine and citalopram significantly prevented the reuptake of 5-HT at an *IC*_50_ of 20.63 μM (*K*_i_ = 6.69 μM) and 18.21 nM (*K*_i_ = 5.9 nM), respectively (Fig. 1L and Table S1). Both the *in vivo* imaging study and *in vitro* data indicate that ketamine directly inhibits SERT to increase cortical 5-HT levels.

### Cryo-EM structure of SERT–ketamine complex in an outward-open conformation

**Figure 2.**
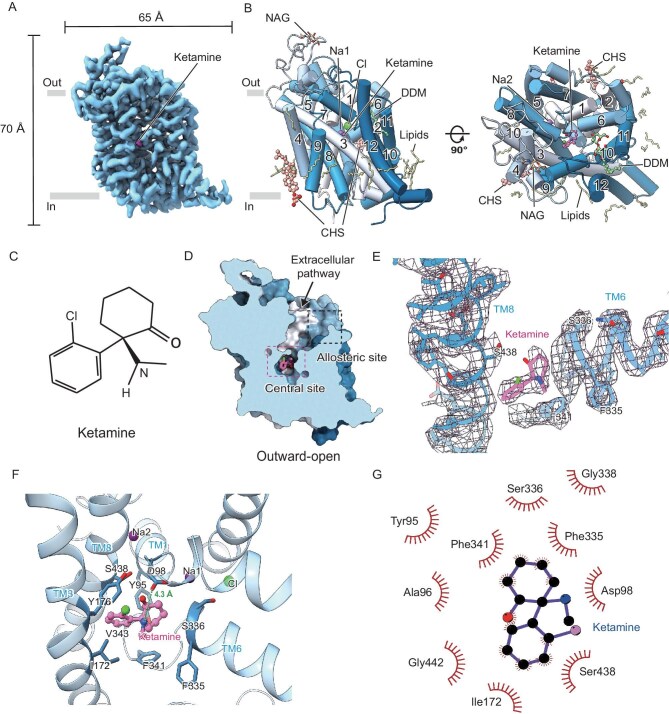
Ketamine binds to the central pocket of hSERT as determined by cryo-EM. (A) Cryo-EM map of ketamine-bound hSERT. (B) Overall structure of hSERT in complex with ketamine. *N*-acetyl glucosamine (NAG; hSERT), *N*-dodecyl-β-d-maltopyranoside (DDM), cholesteryl hemisuccinate (CHS), representative lipids, sodium and chloride ions, and transmembrane helices (TM; 1–12) are labeled. (C) Chemical structure of ketamine. (D) Ketamine (pink stick model) binds to the central pocket of hSERT. (E) Cryo-EM density (mesh) of ketamine (pink stick) and the surrounding TM helices (blue ribbon). (F) Interaction of ketamine in the central pocket. The key residues are shown as sticks. Sodium and chloride ions are shown as balls. (G) LigPlot+ analysis of hSERT^ketamine^.

SERT features a central site and an allosteric site for its substrate 5-HT binding, allowing antidepressants (e.g. paroxetine and citalopram) and psychostimulants (e.g. methamphetamine and cocaine) to occupy one or both of these sites and modulate its transport activity [55,56]. Previous crystal and cryo-EM structures of SERT have revealed that paroxetine, methamphetamine and cocaine bind to the central site, whereas 5-HT and citalopram bind to both sites [55,57–59]. We thus investigated whether ketamine interacts physically with SERT and, if so, which site it targets. We recombinantly expressed human full-length and wild-type SERT (hSERT) with a C-terminal 3C protease site, an enhanced green fluorescent protein (eGFP), and a Strep-tag II in HEK293F cells. Fluorescence-based size-exclusion chromatography (FSEC) analysis of hSERT solubilized in various detergents showed comparable biochemical behavior and molecular weight (Fig. S2A) compared to previously reported data for recombinant human SERT or the native porcine SERT, indicating that the hSERT used in this study is in a homogeneous and monomeric form. We then purified hSERT in the presence of ketamine in detergent micelles and used single-particle cryo-EM to elucidate the structure of the hSERT–ketamine complex (hSERT^ketamine^) at an overall resolution of 3.2 Å (Fig. 2A and B, Fig. S2 and Table S2). A well-resolved ‘Y’-shaped ketamine density in the central site was observed, allowing us to determine the pose and orientation of ketamine (Fig. 2D and E). Moreover, we also observed a detergent molecule of *n*-dodecyl-β-d-maltopyranoside bound to the allosteric site and cholesterols/lipids surrounding the transmembrane region (Fig. 2B), consistent with previous structural and computational studies [58,60].

The overall structure of hSERT^ketamine^ is similar to that of the 5-HT-bound recombinant human SERT and the (+)-methamphetamine-bound or cocaine-bound native porcine SERT, with a root-mean-square deviation (RMSD) value of 0.59, 0.64 and 0.73 Å, respectively (measured from Cα to Cα), indicating that hSERT^ketamine^ adopts an outward-open conformation (Fig. S3A–C). Ketamine lodges between the aromatic groups of Y95, Y176 and F341 (Fig. 2F). The hydrophobic chlorobenzene and cyclohexane groups of ketamine face the lower part of the central site (closer to the cytoplasm), where a hydrophobic barrier is formed by I172, F341, Y95 and V343 (Fig. 2F). The hydrophilic amine group, aryl chloride, and oxygen of ketamine, in contrast, face the upper part of the central site formed by hydrophilic residues D98, S438 and the main chain oxygen of F335 (Fig. 2G). The amine group of ketamine interacts with the carboxylic group of D98, a conserved residue in SERT, at a distance of 4.3 Å (Fig. 2F). Compared with previous structural and functional studies, ketamine occupies a similar but slightly larger volume of the central site than (+)-methamphetamine. In contrast, the larger drugs paroxetine and cocaine almost fill the entire volume (Fig. S3), which may explain, in part, the differences in their binding affinities [58]. Nevertheless, our structural and functional studies of hSERT^ketamine^ show direct binding of ketamine to the central site of SERT.

### Dual inhibition of SERT and NMDAR induces ketamine-like rapid antidepressant effects

**Figure 3.**
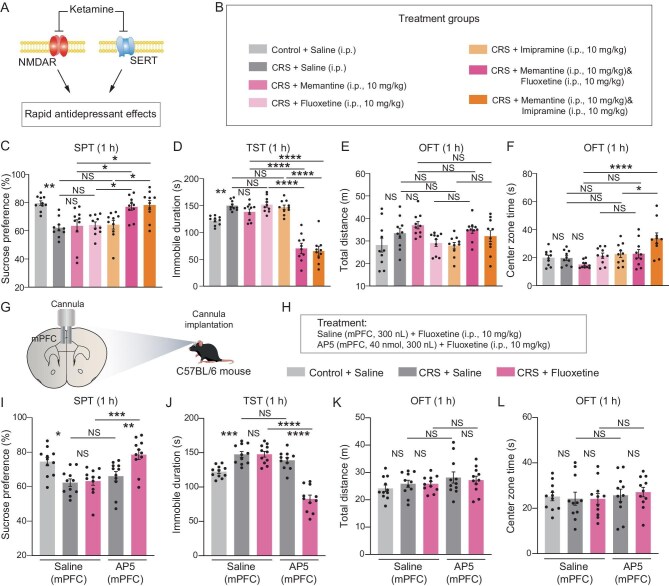
Dual inhibition of SERT and NMDAR induces ketamine-like rapid antidepressant effects. (A) Our proposed mechanism for the dual inhibition of SERT and NMDAR by ketamine. (B) Seven different treatment groups and doses of drugs. CRS, chronic restraint stress. (C–F) Mice in the indicated treatment groups were assessed for sucrose preference with the SPT (C), duration of immobility during the TST (D), total distance traveled (E) and time spent in the center zone (F) during the OFT (10 mice per treatment group). One-way ANOVA with Bonferroni's multiple comparisons test. (G) The site of cannula implantation and drug injection. (H) Three treatment groups and doses of drugs. (I–L) Mice in the indicated treatment groups were assessed for sucrose preference with the SPT (I), duration of immobility during the TST (J), total distance traveled (K) and time spent in the center zone (L) during the OFT (11 mice per treatment group). Two-way ANOVA with Bonferroni's multiple comparisons test. Data are shown as the mean ± SEM. *P < 0.05; **P < 0.01; ***P < 0.001; ****P < 0.0001; NS, not significant (see Table S3 for statistical analyses and *n* numbers).

As ketamine is a non-competitive NMDAR antagonist and several NMDAR antagonists fail to produce rapid antidepressant effects similar to ketamine [17,26,30,34], this suggests that solely inhibiting NMDAR is insufficient to explain the rapid antidepressant effects of ketamine. We hypothesized that the rapid antidepressant action of ketamine may be achieved by simultaneously inhibiting SERT and NMDAR (Fig. 3A). To validate this hypothesis, we used a mouse model of depression that relies on chronic restraint stress [61]. We then intraperitoneally injected the NMDAR antagonist memantine in combination with SERT antagonists fluoxetine or imipramine into mice (Fig. 3B). At 1 h after drug injection, the combined antagonists (memantine in combination with fluoxetine or imipramine) increased the sucrose preference of mice with depression-like symptoms during the sucrose preference test (SPT) (Fig. 3C). During the tail suspension test (TST), the combined antagonists reduced the immobility time of mice with depression-like symptoms (Fig. 3D). Injecting either of the SERT antagonists alone did not, however, produce these effects. These effects were maintained at 24 h after injection of the drugs (Fig. S4A). Furthermore, during the open field test (OFT), the combined use of the NMDAR antagonist and SERT antagonists did not alter the locomotor activity of mice (Fig. 3E). These antagonists alone did not induce anxiety-like behavior in mice, and memantine combined with imipramine increased the activity time of mice in the central zone of the open field (Fig. 3F), indicating a reduction in anxiety-like behavior.

We conducted additional experiments utilizing other NMDAR antagonists, such as MK-801, which failed to replicate ketamine's effects 24 h post-injection in antidepressant-relevant behavioral mouse models [32,62]. These experiments involved administering varying doses of MK-801, both alone and in combination with fluoxetine. Our findings reveal that a single injection of either MK801 or fluoxetine alone does not result in antidepressant effects after 24 h (Fig. S4B and C). In contrast, both 0.03 and 0.1 mg/kg of MK-801 combined with fluoxetine results in lasting antidepressant effects, with 0.03 mg/kg being more effective than 0.1 mg/kg (Fig. S4B and C). However, a combination of 0.01 mg/kg of MK-801 with fluoxetine does not yield a lasting effect. Furthermore, these results showed no significant impact on mouse locomotion and anxiety-like behaviors (Fig. S4D and E).

To further validate whether inhibition of NMDAR in the mPFC combined with SERT inhibition is sufficient to induce rapid antidepressant effects, we again used chronic restraint stress. We injected the NMDAR antagonist AP5 into the mPFC in combination with an intraperitoneal injection of the SERT antagonist fluoxetine in mice (Fig. 3G and H). At 1 h after drug injection, the combined use of both antagonists increased the sucrose preference of mice with depression-like symptoms during the SPT (Fig. 3I). The combined use of both antagonists reduced the immobility time of mice with depression-like symptoms during the TST (Fig. 3J). The combination of AP5 and fluoxetine did not alter locomotor activity or induce anxiety-like behavior in mice during the OFT (Fig. 3K and L). These behavioral results demonstrate that synergic inhibition of SERT and NMDAR induces rapid antidepressant effects similar to those of ketamine.

### Ketamine elevation of 5-HT preferentially activates VIP neurons

**Figure 4.**
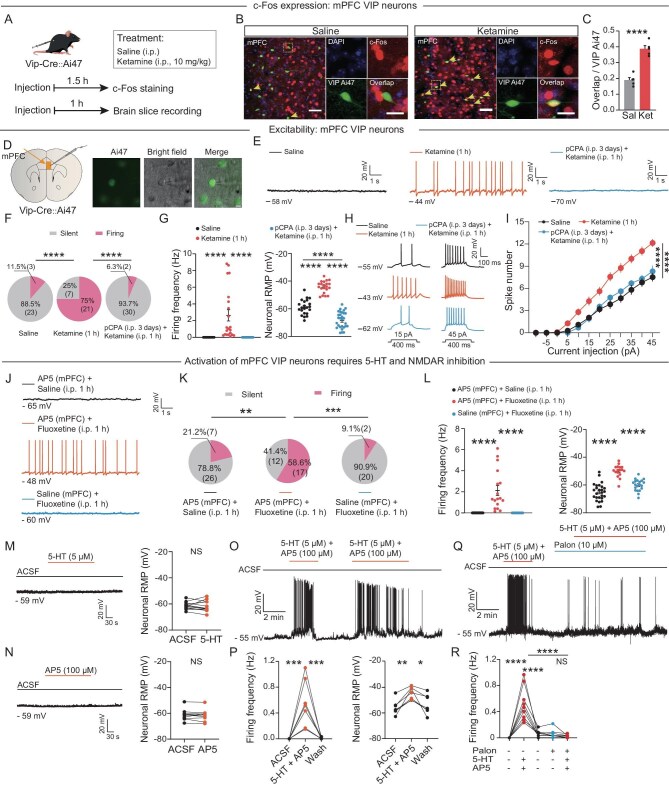
Ketamine activates VIP neurons in the mPFC, which requires 5-HT and requires NMDAR inhibition. (A) Experimental procedure and timeline for determining neuronal activation after ketamine administration in a VIP-Cre::Ai47 mouse. (B) Immunohistochemical staining of c-Fos-expressing neurons in the mPFC at 1.5 h after injection of saline or ketamine. Yellow arrowheads indicate the overlap among c-Fos (red), DAPI (blue) and Ai47 (green, VIP neurons) expression. Scale bar, 50 μm (left), 20 μm (right). DAPI, 4′,6-diamidino-2-phenylindole. (C) Quantitative analysis of c-Fos+ cells among VIP neurons (overlap) in the mPFC (five mice per group). Sal, saline; Ket, ketamine. Two-tailed unpaired *t*-test. (D) Schematic of a brain slice of a VIP-Cre::Ai47 mouse showing the site of injection of ketamine or saline and patch-clamp recording of VIP neurons in the mPFC. (E) Representative traces showing spontaneous activity of VIP neurons 1 h after injection of saline, ketamine (10 mg/kg) or p-CPA (150 mg/kg, once daily for 3 days) with ketamine (10 mg/kg) at the indicated timepoints (four mice per group). p-CPA, DL-4-chlorophenylalanine. (F) Pie charts illustrating the % abundance of silent and firing VIP neurons in (E). Chi-squared test. (G) Quantification of firing frequency (left) and neuronal RMP (right) of neurons as described in (F). RMP, resting membrane potential. Two-tailed unpaired *t*-test. (H) Sample traces of neurons as described in (G). (I) Spike number measurements of neurons as described in (G). Two-way ANOVA with Bonferroni's multiple comparisons test. (J) Representative traces showing spontaneous activity of silent and firing VIP neurons 1 h after AP5 (40 nmol) with saline or AP5 (40 nmol) with fluoxetine (10 mg/kg) or saline with fluoxetine (10 mg/kg) injection. (K) Pie charts illustrate the % abundance of silent and firing VIP neurons in (J). Chi-square test. (L) Quantification of firing frequency (left) and neuronal RMP (right) of neurons as described in (K). Two-tailed unpaired *t*-test. (M and N) Example traces (left; the sample represents 2 min before drug application, 3 min during drug application and 3 min after drug application) and statistics (right) showing the effects of 5-HT (M) and AP5 (N) on mPFC VIP neurons in brain slices from normal mice [13 neurons, 4 mice in (M); 10 neurons, 3 mice in (N)]. Two-tailed unpaired *t*-test. (O) Example trace (sample represents 1 min before drug application, 2 min and 3 min during drug application, and 1.5 min and 2.5 min after drug application) showing the effects of AP5 with 5-HT on mPFC VIP neurons in brain slices from normal mice. (P) Analysis of firing frequency (left) and neuronal RMP (right) of neurons as described in (O) (seven neurons, three mice). Two-tailed unpaired *t*-test. (Q) Example trace (sample represents 1 min before and 2 min and 3 min during drug application and 1.5 min and 2.5 min after drug application) showing the effects of AP5 with 5-HT before and after palon application on mPFC VIP neurons in brain slices from normal mice. Palon, palonosetron. (R) Analysis of firing frequency (left) of neurons as described in (Q) (nine neurons, three mice). One-way ANOVA with Bonferroni's multiple comparisons test (R). Data are shown as the mean ± SEM. *P < 0.05; **P < 0.01; ***P < 0.001; ****P < 0.0001; NS, not significant (see Table S3 for statistical analyses and *n* numbers).

VIP, parvalbumin (PV) and somatostatin (SST) neurons are the three main types of gamma-aminobutyric acid (GABA)ergic interneurons in the mPFC [63]. Inhibition of PV and SST neurons is involved in the rapid antidepressant effects of ketamine [51–53], but the role of VIP neurons in the effects of ketamine has not been studied. To investigate the effects of ketamine on these interneuron types in the mPFC, we labeled VIP, PV and SST neurons by crossing Ai47 (Cre-dependent GFP reporter) mice with VIP-Cre, PV-Cre and SST-Cre mice [64]. Immunolabeling for c-Fos in mice expressing GFP in specific neuronal populations showed that ketamine specifically activated VIP neurons at the antidepressant dose (38.8% of neurons activated on average) in the mPFC (Fig. 4A–C), but PV and SST neurons were not activated (Fig. S5A–F).

To investigate whether ketamine changes the electrophysiological characteristics of VIP neurons in the mPFC, we then carried out patch-clamp recordings of the identified mPFC VIP neurons in mice 1 h after injection of ketamine (Fig. 4D). The percentage of firing VIP neurons increased significantly from 11.5% (3 of 26 cells firing) in controls to 75% (21 of 28 cells firing; 1 h after injection) in the ketamine group (Fig. 4E and F). In addition, the resting membrane potential (RMP) of VIP neurons was, on average, more depolarized (−44.2 mV) after ketamine injection compared with that of saline controls (−58.8 mV) (Fig. 4G). These neurons also exhibited a remarkable increase in the number of spikes in response to depolarizing current pulses (Fig. 4H and I). Interestingly, these firing neurons showed an increase in neuronal membrane resistance with no change in membrane capacitance (Fig. S6A and B). In contrast, neither PV nor SST neurons showed any changes in electrophysiological characteristics of excitability (Fig. S6C–L). Thus, ketamine specifically elevates the excitability of mPFC VIP neurons.

Traditional antidepressants that target the serotonergic system to increase brain levels of 5-HT have long been used to treat psychiatric disorders [4,5]. To examine whether 5-HT is necessary for the rapid antidepressant effects of ketamine, we intraperitoneally injected the 5-HT hydroxylase inhibitor 4-chloro-dl-phenylalanine (p-CPA) three times, with one injection per day, to decrease the levels of 5-HT (Fig. S7A). p-CPA attenuated the ketamine-related decrease in the duration of immobility during the TST and forced swimming test (FST) (Fig. S7B, C) but did not affect locomotion or cause anxiety-like behaviors during the OFT at 1 h after injection (Fig. S7D and E). Meanwhile, activation of mPFC VIP neurons by ketamine was blocked after the p-CPA injection (Fig. 4E and F) and the RMP of VIP neurons was, on average, more hyperpolarized (−69.7 mV) after p-CPA relative to saline controls (−58.8 mV) (Fig. 4G). The number of spikes in response to depolarizing current pulses also decreased after the p-CPA injection relative to slices treated with ketamine alone (Fig. 4H and I). These results indicate that ketamine specifically activates mPFC VIP neurons and that this activation requires 5-HT.

### The synergistic effect of 5-HT and NMDAR inhibition activates VIP neurons

To assess whether the elevation of 5-HT alone could activate mPFC VIP neurons, we intraperitoneally injected the SERT inhibitor fluoxetine into the VIP-Cre mice to increase the 5-HT level. Fluoxetine alone did not increase the excitability of mPFC VIP neurons relative to saline controls (Fig. 4J–L). Consistently, the application of 5-HT (5 μM) to brain slices also did not activate VIP neurons in normal mice (Fig. 4M). To further assess whether inhibition of NMDAR directly activates mPFC VIP neurons, we carried out an identical set of electrophysiological experiments and made whole-cell recordings of mPFC VIP neurons in brain slices from normal mice after application of the NMDAR inhibitor AP5 (100 μM). Notably, the direct application of AP5 did not activate VIP neurons (Fig. 4N). Thus, inhibition of NMDARs alone is not sufficient to activate VIP neurons.

To further assess the roles of SERT and NMDAR in the activation of mPFC VIP neurons, AP5 (40 nmol) was injected into the mPFC in conjunction with an injection of fluoxetine. This increased the firing of some VIP neurons (58.6%, Fig. 4J and K), and the RMPs of these VIP neurons were, on average, more depolarized (−49.8 mV) than those of neurons after treatment with AP5 and saline (–65.1 mV) (Fig. 4L). Importantly, bath application of 5-HT (5 μM) and AP5 (100 μM) together induced the firing of some VIP neurons (Fig. 4O) and significantly increased the RMP of these VIP neurons (Fig. 4P). These results indicate that activation of VIP neurons requires the synergistic effect of elevated 5-HT and NMDAR inhibition together.

### VIP neurons receive 5-HT inputs from the dorsal raphe nucleus

To investigate whether there is a specific connection between mPFC VIP neurons and 5-HT neurons, we applied a rabies virus (RV)-mediated trans-synaptic retrograde tracing strategy to map the afferent inputs of the mPFC VIP neurons (Fig. S8A). After 3 weeks, the mice were perfused, and their brains were processed for subsequent data analysis (Fig. S8B). Initially infected cells (expressing both GFP and DsRed) and retrogradely labeled RV neurons (expressing DsRed alone) were identified (Fig. S8C). RV-tracing results showed that DRN 5-HT neurons project onto VIP neurons (Fig. S8D). These results identify that serotonergic signaling is involved in the ketamine-induced increase in the excitability of mPFC VIP neurons.

### 5-HT signaling mediates the activation of VIP neurons by ketamine and the rapid antidepressant effects of ketamine

The data above indicate that serotonergic signaling is important for the activation of mPFC VIP neurons. However, the pathways through which VIP neurons mediate the effects of 5-HT signaling are not clear. Morphological and electrophysiological results have indicated that VIP neurons are the 5-HT3A receptor (5-HT3AR) interneurons in the neocortex [63], and ketamine can potentiate 5-HT3AR-mediated currents in ganglion neurons [65]. We thus determined whether the 5-HT3AR antagonist palonosetron, when injected into the mPFC, can prevent the enhanced excitability of mPFC VIP neurons induced by ketamine. To do so, we performed whole-cell recordings of mCherry-labeled VIP neurons in the mPFC (Fig. S9A). The percentage of firing VIP neurons was ∼10%, which was almost equal to the controls (∼10.5%) (Fig. S9B). Consistently, blocking 5-HT3AR also blocked the effect of 5-HT combined with AP5 in activating VIP neurons (Fig. 4Q and R). Furthermore, there was no change in the RMP (Fig. S9C). There was no change in membrane resistance (Fig. S9D) and a moderate increase in membrane capacitance (Fig. S9E) among VIP neurons. Next, we tested whether the rapid antidepressant effects of ketamine on mPFC VIP neuron activation require 5-HT3AR. We injected palonosetron or saline bilaterally into the mPFC 0.5 h before the injection of ketamine (Fig. S9A and F). Mice that did not receive palonosetron had a shorter duration of immobility during the TST and FST at 1 h and 24 h after injection of ketamine compared with control mice, but this effect was blocked by palonosetron (Fig. S9G–J). In addition, injection of palonosetron into the mPFC did not affect locomotion or cause anxiety-like behaviors during the OFT at 1 h after injection (Fig. S9K and L). To further verify whether directly interfering with 5-HT3AR expression in VIP neurons can block the effects of ketamine, we used RNA-specific knockdown of 5-HT3AR expression in the mPFC VIP neurons of VIP-Cre mice (Fig. S10A). Consistent with the results of the blocker, there was no difference in the immobility time in the TST of ketamine-treated mice compared to the control group (Fig. S10C), and the SPT did not show a significant increase (Fig. S10B). Moreover, the mice's motor abilities and anxiety-like behaviors were not affected (Fig. S10D and E).

Therefore, ketamine directly inhibits SERT to increase the levels of 5-HT in combination with inhibition of the NMDAR to activate mPFC VIP neurons. Furthermore, the blockade of 5-HT3AR prevents the ketamine-induced increase in the excitability of VIP neurons in the mPFC, which ultimately blocks the antidepressant effects of ketamine.

### Activation of mPFC VIP neurons is necessary for rapid antidepressant effects

**Figure 5.**
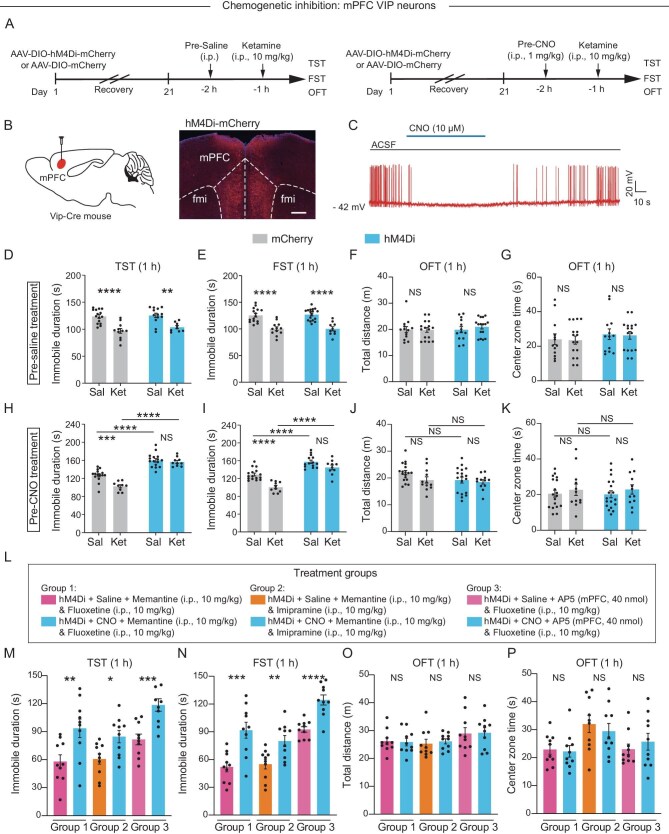
Activation of mPFC VIP neurons is necessary for the rapid antidepressant effects. (A) Experimental procedure and timeline. Mice were pre-treated with either saline (Pre-saline) or CNO (Pre-CNO) 1 h before injection with saline or ketamine. (B) Chemogenetic manipulation (left) and example site of hM4Di expression (right) in the mPFC of VIP-Cre mice. Scale bar, 200 μm. (C) A representative trace showing action potential inhibition after CNO application during cell-attached recording in mPFC VIP neurons. (D–K) hM4Di and mCherry mice pre-treated with either saline (D–G) or CNO (H–K) were then injected with either saline or ketamine and were later assessed for the duration of immobility during the TST (D and H) and the FST (E and I) and total distance traveled (F and J) and time spent in the center zone (G and K) during the OFT. Sal, saline; Ket, ketamine; fmi, forceps minor of the corpus callosum. Two-way ANOVA with Bonferroni's multiple comparisons test. (L) Three different treatment groups and doses of drugs. (M–P) hM4Di mice pre-treated with either saline or CNO were then injected with one of three treatments and were later assessed for the duration of immobility during the TST (M) and the FST (N) and the total distance traveled (O) and center zone time (P) during the OFT (10 mice per group). Two-tailed unpaired *t*-test. Data are shown as the mean ± SEM. *P < 0.05; **P < 0.01; ***P < 0.001; ****P < 0.0001; NS, not significant (see Table S3 for statistical analyses and *n* numbers).

To determine whether VIP neurons in the mPFC are necessary for the rapid antidepressant effects of ketamine, we performed chemogenetic inhibition and genetic ablation experiments. In the chemogenetic inhibition assay, we injected the AAV-DIO-hM4Di-mCherry vector into the bilateral mPFC of VIP-Cre mice. Subsequent expression of the hM4Di receptor allowed inhibition of VIP neurons (Fig. 5A and B, Fig. S11A–F). Specifically, the application of clozapine *N*-oxide (CNO, 10 μM), a specific ligand of the hM4Di receptor, significantly inhibited mPFC VIP neuron activity in brain slices (Fig. 5C). Behavioral experiments were performed after mice had been allowed to recover for 3 weeks after viral injection (Fig. 5A). Ketamine injected 1 h after the initial saline treatment decreased the time that the mice were immobile during the TST and FST (Fig. 5D and E). However, chemogenetic inhibition of mPFC VIP neurons by CNO injection increased the duration of immobility of the mice during the TST and FST (Fig. 5H and I). The effects of ketamine were blocked after inhibition of mPFC VIP neurons (Fig. 5H and I). These findings are consistent with results at 24 h after injection of ketamine (Fig. S12A–D). Furthermore, chemogenetic inhibition of mPFC VIP neurons did not affect locomotion or anxiety-related behaviors during the OFT (Fig. 5F, G, J and K).

To further examine whether ablation of mPFC VIP neurons would alter the effects of ketamine, we injected the vector AAV-DIO-taCasp3-TEVp or the control vector AAV-DIO-GFP bilaterally into the mPFC of VIP-Cre mice. Behavioral experiments were performed after mice had been allowed to recover for 4 weeks after viral injection. Ablation of mPFC VIP neurons decreased sucrose preference during the SPT and increased the duration of immobility during the TST and FST (Fig. S12E–G). Similarly, the ablation of mPFC VIP neurons blocked the antidepressant effect of ketamine (Fig. S12E–G). Ablation of mPFC VIP neurons did not change the locomotor activity or anxiety-like behaviors of these mice during the OFT (Fig. S12H, I), which indicated that the change in immobility was not likely due to motor defects. Meanwhile, no obvious changes were observed in measurements of body weight or water consumption (Fig. S12J, K) or in a social interaction test (Fig. S12L, M). Therefore, the activity of mPFC VIP neurons signals mood states and is important in mediating the rapid antidepressant effects of ketamine.

We show above that the combined use of SERT inhibitors (fluoxetine or imipramine) and NMDAR inhibitors (memantine or AP5) can produce rapid antidepressant effects similar to those of ketamine. To validate whether inhibiting VIP neurons can also block these effects, we performed chemogenetic inhibition experiments (Fig. 5M). The combined drug administration reduced the baseline immobility time in control mice. However, inhibiting mPFC VIP neurons substantially blocked the alterations in immobility time caused by the combined drug administration during the TST and FST (Fig. 5M and N). These findings are consistent with results at 24 h after the combined drug administration (Fig. S4B). Similarly, chemogenetic inhibition of mPFC VIP neurons did not affect locomotion or anxiety-related behaviors during the OFT (Fig. 5O and P). These results indicate that activation of mPFC VIP neurons by dual inhibition of both NMDAR and SERT is key for the rapid antidepressant effects of ketamine.

## DISCUSSION

Our results provide novel insights into the cellular mechanisms that underlie ketamine's rapid antidepressant effects, with promising implications for devising novel treatments for major depressive disorder. We show that ketamine exerts its effects through a dual-action mechanism: it inhibits SERT, thereby elevating 5-HT levels, while also blocking NMDAR. Furthermore, dual inhibition of SERT and NMDAR mimics ketamine-like rapid antidepressant effects, and the serotonin-dependent activation of VIP neurons governs the rapid antidepressant effects. Thus, our findings suggest that this dual-action mechanism is essential for the rapid antidepressant effects of ketamine.

Our data demonstrate that elevating 5-HT levels via SERT inhibition plays a pivotal role in ketamine's rapid antidepressant effects. Decreased cortical 5-HT is well recognized as a notable pathological feature of depression [4,42]. Traditional antidepressant drugs such as SSRIs work by increasing extracellular 5-HT levels through the inhibition of SERT, but they typically take a long time to become effective [4,5]. This suggests that merely increasing extracellular 5-HT levels is not sufficient to produce rapid antidepressant effects. Through various interdisciplinary techniques, we reveal that ketamine can directly bind to the central pocket of SERT and stabilize it in an outward-open state, thereby inhibiting the SERT transport of extracellular 5-HT, which in turn increases the concentration of extracellular 5-HT, rather than by activating 5-HT neurons in the DRN. However, unlike SSRIs, when ketamine inhibits SERT, it also simultaneously inhibits NMDARs, which represents a previously unreported synergistic effect. Furthermore, reducing the synthesis of 5-HT impairs the rapid antidepressant effect of ketamine, indicating that the rapid antidepressant actions of ketamine are dependent on 5-HT (Fig. S7).

In addition, we find that the inhibition of SERT and NMDAR by ketamine activates VIP neurons rather than other interneurons (PV and SST neurons) in the mPFC. VIP neurons in the mPFC serve as an intriguing focal point in this mechanism. These neurons influence emotional and social behaviors by targeting other neurons and facilitating the disinhibition of pyramidal neurons [66–68]. Moreover, our data suggest a specific link between DRN 5-HT neurons and mPFC VIP neurons (Fig. S8). Although 5-HT alone does not activate VIP neurons, its effect becomes significant when combined with NMDAR inhibition, indicating a synergistic relationship. Recent studies have shown that cortical VIP neurons are inhibited by interneurons [69–71]. One proposed mechanism is that NMDAR blockade may potentiate the effect of 5-HT on VIP neurons by inhibiting NMDARs in other interneurons, thereby disinhibiting VIP neurons. Simultaneously, SERT inhibition increases 5-HT levels, which could synergistically boost VIP neurons’ excitability through 5-HT3AR, ultimately leading to the activation of VIP neurons. This activation, driven by the inhibition of SERT and NMDAR, is essential for the rapid antidepressant effects of ketamine.

Clinical evidence indicates that traditional antidepressants, such as SSRIs, typically require 2 weeks or longer to take effect, which can lower medication compliance among depressed patients [4,5,72,73]. In contrast, ketamine has been shown to take effect rapidly within hours and can last up to a week, delivering a fast-acting antidepressant effect, even for patients with treatment-resistant depression who do not respond to SSRIs [8,74,20]. Significantly, recent clinical studies have demonstrated that the co-administration of esketamine with SSRIs produces enhanced therapeutic effects compared to either treatment alone, supporting the presence of synergistic interactions in clinical combination therapy that improve antidepressant efficacy [75–77]. Consistent with these findings, this study shows that memantine, an NMDAR antagonist without intrinsic antidepressant properties, when given in combination with SSRIs, produces significant and rapid antidepressant effects, thereby confirming the therapeutic potential of this clinical combination strategy.

Hydroxynorketamine (HNK), a metabolite of ketamine, has recently been identified as having rapid and sustained antidepressant effects [78–82], marking a significant advancement in psychiatric pharmacology. Mechanistically, recent studies suggest that HNK likely exerts its antidepressant effects through molecular pathways rather than NMDAR inhibition [32,78,80,83–86]. These insights reveal that ketamine's rapid antidepressant effects involve the synergistic inhibition of both SERT and NMDAR, rather than solely NMDAR inhibition, distinguishing its mechanism from that of HNK. However, further research is needed to elucidate the mechanisms underlying HNK's rapid and sustained antidepressant effects. Additionally, there is also evidence that NMDAR-activation-dependent long-term potentiation (LTP)-like mechanisms may contribute to ketamine's antidepressant effects [62]. It is well known that LTP is modulated by the local balance of excitatory and inhibitory inputs, and interneurons are involved in this process [87–90]. VIP neurons may contribute to circuit disinhibition by suppressing other inhibitory interneurons, thereby facilitating pyramidal neuron activation and enhancing synaptic plasticity [68,91–96]. It remains to be investigated whether the dual inhibition of SERT and NMDAR influences synaptic plasticity-related proteins, thereby contributing to ketamine's long-term antidepressant effects.

Whether ketamine at a rapid-acting antidepressant dose binds to SERT remains a critical question. Our data show that ketamine, at such a dose, can reach brain concentrations exceeding 10 μM, consistent with previous reports [19,22,32]. Although some studies have described minimal or weak binding of ketamine to SERT [96–98], this discrepancy is likely due to the limited concentration range tested—typically capped at 10 μM—which is insufficient to fully characterize ketamine's binding affinity within the micromolar range. In conclusion, our research opens up a new avenue for understanding the molecular and cellular basis of ketamine's rapid antidepressant effects. By pinpointing the dual inhibition of both SERTs and NMDARs as a key mechanism, we lay the groundwork for the development of novel, more efficacious treatments for major depressive disorder. Our findings emphasize the importance of a synergistic approach to neurotransmitter modulation and set the stage for future research.

## MATERIALS AND METHODS

### Animals

The mice were group-housed (four to five mice per cage) under a 12 h light/dark cycle (light on from 7:00 A.M. to 7:00 P.M.), with *ad libitum* free access to water and food at 22°C–25°C with 40%–60% relative humidity. VIP-Cre (JAX), PV-Cre (JAX), SST-Cre (JAX), ePet-Cre (JAX), Ai47 (JAX) and C57BL/6 mice (Shanghai Jihui Laboratory Animal Breeding Co.) were used. Transgenic mice were bred in Shanghai Model Organisms Center, Inc. VIP-Cre::Ai47 mice were derived from crosses of the VIP-Cre and Ai47 genotypes. PV-Cre::Ai47 mice were derived from crosses of the PV-Cre and Ai47 genotypes. SST-Cre::Ai47 mice were derived from crosses of the SST-Cre and Ai47 genotypes. Male adult (8–16 weeks of age) mice were used for the experiment and were randomly selected. All animals were habituated for 1 week before the experiments. All experimental procedures were approved by the Institutional Animal Care and Use Committee at Fourth Military Medical University and performed according to National Institutes of Health guidelines.

### mPFC slice preparation

Adult (10–16 weeks old) VIP-Cre-expressing mCherry mice with or without a cannula were firstly anesthetized by being injected intraperitoneally with tribromoethanol (250 mg/kg) and then perfused transcardially with 20 mL of ice-cold *N*-methyl-d-glucamine (NMDG) artificial cerebrospinal fluid (ACSF) solution (oxygenated with 5% CO_2_ + 95% O_2_) containing (mM): 93 NMDG, 93 HCl, 2.5 KCl, 10 MgSO_4_·7H_2_O, 1.2 NaH_2_PO_4_·2H_2_O, 30 NaHCO_3_, 25 glucose, 20 HEPES, 5 sodium ascorbate, 3 sodium pyruvate and 2 thioureas, with 0.5 CaCl_2_·2H_2_O added (pH: 7.35, 303 mOsm). The brain was removed quickly after decapitation and immediately put into ice-cold oxygenated NMDG ACSF. A few minutes later, coronal slices containing the mPFC (300 μm thickness for mice) were sectioned in cold ACSF using a vibratome (VT1200 S, Leica) and then transferred into oxygenated NMDG ACSF at 32°C for incubation and recovery for 10–15 min. The brain slices were transferred to a normal oxygenated ACSF solution (126 mM NaCl, 2.5 mM KCl, 2 mM MgSO_4_·7H_2_O, 1.25 mM NaH_2_PO_4_·2H_2_O, 25 mM NaHCO_3_, 10 mM glucose, 2 mM CaCl_2_) at room temperature for 1 h. All chemicals were purchased from Sigma–Aldrich (St. Louis, MO, USA).

### Statistical analysis

The images of brain sections were processed by ImageJ (NIH, USA) software and QuPath (University of Edinburgh, Scotland). The statistical analyses were conducted using GraphPad Prism software v10.0 (GraphPad, USA) and MATLAB R2023b (MathWorks, USA) software. All statistical tests were two-tailed, and significance was considered at *P < 0.05, **P < 0.01, ***P < 0.001, ****P < 0.0001. When sample groups had normality and equal variance, data were analyzed by paired or unpaired *t*-test and one-way or two-way analysis of variance (ANOVA) (followed by Bonferroni's multiple comparisons test). The difference in the proportion of firing was analyzed using the chi-squared test. The experimental data retain two significant figures, expressed as mean ± standard error of the mean (mean ± SEM). The details of sample sizes, statistical tests used, *P* value and treatment effect of our statistical analyses for each experiment are described in Table S3.

## Supplementary Material

nwaf367_Supplemental_File

## Data Availability

The data to evaluate the conclusions of this study are available within the article and the Supplementary data. Additional data are available upon request.
