# Supplementary material for: Inhibition of SERT and NMDAR synergistically confers rapid antidepressant effects of ketamine
Source: Natl Sci Rev. 2025 Sep 8;12(12):nwaf367. doi: 10.1093/nsr/nwaf367 (PMC12707061; doi:10.1093/nsr/nwaf367)
Supplement: nwaf367_Supplemental_File [file nwaf367_supplemental_file.docx]

**MATERIALS AND METHODS**

**Viral vectors and stereotaxic surgery**

All recombinant adeno-associated viruses (rAAVs) used in this study included: rAAV-Ef1α-DIO-mCherry-WPRE-hGH polyA (titre: 5.0 x 10^12^ v.g./mL, 0.2 µL bilateral into mPFC) rAAV-hSyn-DIO-hM4D(Gi)-mCherry-WPRE-hGH polyA (titre: 5.25 x 10^12^ v.g./mL, 0.2 µL bilateral into mPFC), rAAV-Ef1α-DIO-EGFP-WPRE-hGH polyA (titre: 5.0 x 10^12^ v.g./mL, 0.2 µL bilateral into mPFC) and rAAV-Ef1α-flex-taCasp3-TEVp-WPRE-hGH polyA (titre: 5.09 x 10^12^ v.g./mL, 0.2 µL bilateral into mPFC) were purchased from the BrainVTA (Wuhan, China). For surgery, mice were anesthetized with a mixture of isoflurane (1.0-1.5%) and oxygen (0.6-0.8 L/min), positioned in a stereotaxic instrument (RWD, Wuhan, China) with an electric heating pad (BrainKing Biotech) to keep mice warm (37 °C) and the skull surface was exposed. All surgical tools and materials were sterilized with alcohol. AAV virus was infused (at a rate of 40 nL/min) into either unilateral or bilateral mPFC (coordinates: AP, +1.65 mm from the bregma; ML, ±0.25 mm from sagittal structure; DV, –1.60 mm from dura) or DRN (coordinates: AP, +4.60 mm from the bregma; ML, ±0.05 mm from sagittal structure; DV, –2.80 mm from dura) by using a micro-syringe pump (Nanoject III #3-000-207, DRUMNOND). Needles were slowly withdrawn 10 min after the injection was finished.

For retrograding trace experiments, rAAV-Ef1α-DIO-H2B-EGFP-T2A-TVA-WPRE-hGH polyA (titre: 5.0 x 10^12^ v.g./mL, 0.2 µL unilateral into mPFC) and rAAV-Ef1α-DIO-RVG-WPRE-hGH polyA (titre: 5.0 x 10^12^ v.g./mL, 0.2 µL unilateral into mPFC) helper viruses were injected into the unilateral mPFC (coordinates: AP, +1.65 mm from the bregma; ML, ±0.25 mm from sagittal structure; DV, –1.60 mm from dura), after two weeks, RV-EnVA-△G-DsRed (titre: 2.0 x 10^8^ IFU/mL, 0.3 µL unilateral into mPFC) was injected into the same location.

For RNA-specific knockdown experiments, rAAV-Ef1α-DIO-Luciferase-EGFP-WPRE-

hGH polyA (titre: 3.0 x 10^12^ v.g./mL, 0.2 µL unilateral into mPFC) and rAAV-Ef1α-DIO-Scramble-EGFP-WPRE-hGH polyA (titre: 3.0 x 10^12^ v.g./mL, 0.2 µL unilateral into mPFC) and rAAV-Ef1α-DIO-5HT3AR (shRNA)-EGFP-WPRE-hGH polyA (titre: 3.0 x 10^12^ v.g./mL, 0.2 µL unilateral into mPFC) were injected into the unilateral mPFC (coordinates: AP, +1.65 mm from the bregma; ML, ±0.25 mm from sagittal structure; DV, –1.60 mm from dura), after four weeks, behavioral experiments were performed.

For *in vitro* electrophysiological recordings, AAV-Ef1α-DIO-mCherry was delivered bilaterally into the mPFC of male Vip-Cre mice or the DRN of male ePet-Cre mice. For the chemogenetic experiment, AAV-Syn-DIO-hM4D(Gi)-mCherry, AAV-CAG-DIO-taCasp3-TEVp, AAV-Ef1α-DIO-mCherry, or AAV-CMV-DIO-EGFP was delivered bilaterally into the mPFC of male Vip-Cre mice, respectively. For the microinjection experiment, a 26-gauge stainless steel cannula (3.0 mm in length from the cannula base) was implanted bilaterally into the mPFC (coordinates: AP, +1.65 mm from the bregma; ML, ±0.35 mm from sagittal structure; DV, –1.50 mm from dura).

**Systemic drug delivery**

All drugs were dissolved in 0.9% saline and administered by intraperitoneal (i.p.) injection. Ketamine (10 mg/kg, Gutian Pharma Co.), Memantine (10 mg/kg, APEX), MK-801 (0.01, 0.03, 0.1 mg/kg, APEX), fluoxetine (10 mg/kg, Sigma Aldrich), and imipramine (10 mg/kg, Sigma Aldrich) for chemogenetic experiments. Ketamine (10 mg/kg) and CNO (1 mg/kg, Sigma Aldrich) for the chemogenetic experiment. Ketamine (10 mg/kg), AP5 (40 nmol, Sigma Aldrich), and fluoxetine (10 mg/kg, Sigma Aldrich) for slice recording, mice were used for behavioral studies or killed for *in vitro* electrophysiology studies.

**Chemogenetic manipulation**

Behavioral experiments were performed after 3-4 weeks of viral expression. For the inhibition experiment, intraperitoneal injection of CNO (1 mg/kg) was performed for 1 h before ketamine (10 mg/kg) was intraperitoneally injected, and the corresponding behavioral tests were performed on the next 1 h and 24 h, respectively, after injection of ketamine. For the ablation experiment, ketamine (10 mg/kg) was intraperitoneally injected, and the antidepressant behavioral tests were performed the next day.

**Cannula microinjection procedures**

For behavioral experiments, C57BL/6 mice were recovered for at least 7 days before behavioral testing. Before micro-infusions, a 26-gauge internal injector with 3 mm projection was placed into the guide cannula, connected via PE tubing to a Hamilton micro-syringe driven by hand. Ketamine (25 µg, 300 nL, bilaterally) or palonosetron (10 µg, 300 nL, bilaterally) (Sigma Aldrich) or 0.9% saline (300 nL, bilaterally) was administered at a rate of 200 nL/min through an injector, and the injector was left in situ for an additional minute to prevent backflow. The injector cannula was removed 3 min after infusion was finished, and corresponding behavioral tests were performed.

For *in intro* brain slices recording, AAV-Ef1α-DIO-mCherry virus was injected in the mPFC for labeling VIP neurons, and a 26-gauge double guide cannula (center-to-center distance 0.80 mm; RWD, China) was subsequently implanted in the same site of Vip-Cre mice. After 3 weeks of virus expression and recovery, ketamine was injected intraperitoneally for 0.5 h after palonosetron, or saline was infused by an injector. After 1 h of ketamine injection, mice were killed for electrophysiological recording.

**Behavioral assays**

All behavioral experiments were performed between 4:00 P.M. and 9:00 P.M., and mice were 10-16 weeks old for behavioral tests. FST and TST were performed during the light phase (4:00 P.M. to 7:00 P.M.), these behavioral experiments were performed in normal light conditions (100 Lux); SIT, OFT, and SPT were performed during the dark phase (7:00 P.M. to 9:00 P.M.), these tests were conducted under dim light conditions (3–5 Lux). Behavioral data were analyzed by researchers blinded to the experiments.

**Chronic restraint stress (CRS) paradigm**

The protocols of CRS were designed to maximize the unpredictable factor of the stressor. CRS procedures were performed as described previously with minor modifications [61]. Mice for the model were singly housed and received 2 weeks of restraint stress. Model mice were restrained in a 50 mL tube for 2-3 h. Control mice were group-housed without stress administration. All mice lived in inconsistent environmental conditions. After the CRS procedure, the following day, mice performed a battery of behaviors in the order of TST, FST, SPT, and OFT, with at least 2 h resting time between tests. SPT, TST, and FST were carried out on the second day after a single administration of the putatively effective fraction.

**Sucrose preference test (SPT)**

For the adaptive phase, mice were individually housed and exposed to a bottle of sucrose solution (2% in distilled water) and accompanied by a bottle of water for 72 h, followed by 24 h of water deprivation. For the test phase, mice were exposed to a 2 h exposure to two identical bottles, one filled with 2% sucrose solution and the other with water. The volume of sucrose solution or water was measured after 2 h of exposure. The positions of the two bottles were switched every 24 h in the adaptation period and after 1 h in the test period. Sucrose preference was defined as the ratio of the sucrose consumption weight versus the total consumption weight of sucrose and water during the 2 h test.

**Tail suspension test (TST)**

The mice were suspended in a computerized device allowing four animals to be recorded at one time, and were separated in a chamber, both acoustically and visually isolated. To ensure mice could not contact or climb with other things, an individual mouse was suspended 50 cm above the floor by a tail tape that was placed approximately 1 cm from the tip of the tail. The activities of the animals were recorded for 6 minutes by a video recorder. The total duration of immobility at the last 4 minutes of testing time was analyzed by one who was blinded to the test.

**Forced swimming test (FST)**

The FST apparatus was a clear glass tank (40 cm high and 20 cm in diameter) filled with water (25-26 °C), and the water depth was set to ensure mice could not touch the bottom with their tails or hind limbs. Mice were placed individually into the apparatus and allowed to swim for 6 minutes. The mice were considered immobile when floating in the water without struggling and making only those movements necessary to keep their heads above the water. Total immobility times during the last 4 minutes of the 6-minute testing period were analyzed by a researcher blinded to the test.

**Open field test (OFT)**

Mice were placed in a white open field chamber (38 x 38 x 40 cm) built by an acrylic plate in a room with dim light. The movement of each animal was tracked by a video camera positioned directly above the chamber (Jiliang, Shanghai). Time spent in the center zone of the chamber was a measure of anxiety behavior. Mice were allowed to move freely for 5 minutes in the arena. Total distance and center zone time were automatically analyzed by software (Jiliang, Shanghai).

**Social interaction test (SIT)**

Each test mouse was placed in a two-chamber (50 x 25 x 35 cm) in a room with dim light. One empty wire mesh cup was placed in one of the chambers, and another wire mesh cup was placed in the other chamber with a conspecific mouse. The movement of each test animal was tracked by a video camera positioned directly above the chamber (Jiliang, Shanghai). Interaction time was calculated as the time during the test mouse explored the conspecific mouse. Test mice were allowed to move freely for 5 minutes in the arena.

***In vitro* electrophysiological recordings**

For mPFC VIP neuron recordings, brain slices were transferred into the recording chamber filled with ACSF. VIP positive neurons were visualized with differential interference contrast optics (DIC, Olympus BX61WI). The recording pipettes (4-5 MΩ) were pulled with a micropipette puller (P97, Sutter Instrument; USA). For whole-cell patch-clamp recording, the pipette was filled with an internal solution containing (mM) 133 potassium gluconate, 18 NaCl, 0.6 EGTA, 2 Mg·ATP, 0.3 Na_3_·GTP, 10 Hepes (pH: 7.20, 280-290 mOsm). The MultiClamp 700B amplifier and pCLAMP10 software (Axon Instruments, USA) were used for all recordings with signals that were low-pass filtered at 2 kHz and digitized at 10 kHz (DigiData 1550, Molecular Devices). When the whole-cell record was formed, to record the neuronal firing, the neurons were held at *I*=0 pA under current-clamp mode; to measure the intrinsic membrane properties, the same neurons were held at *V*= –70 mV under voltage-clamp mode and spikes were induced by increases of current injection (each step was 5 pA, range –10-45 pA). All recordings were performed for 5 minutes typically.

To investigate the underlying synergistic effects of 5-HT (5 μM, Sigma) and AP5 (100 μM, Sigma) on neuronal excitability and firing, the two drugs were alone or together continuously perfused into the recording chamber using a medicine delivery pipe after a baseline was recorded for 5 min. The spikes were recorded under voltage-clamp mode 1 minute after the drugs were delivered. To test the effects of palonosetron (10 μM, Sigma) on firing induced by 5-HT and AP5, firstly, we performed 5-HT and AP5 to induce neuronal firing occurred then washed for 5 minutes, then palonosetron was continuously perfused into the recording chamber using a delivery pipe. After a few minutes for stabilization, 5-HT and AP5 were again perfused for at least 5 minutes.

**Slice data analysis**

Electrophysiological data were analyzed using Clampfit 10.7 (Molecular Devices, USA) and MATLAB R2023b (Mathworks, USA) software. The number of action potentials is automatically measured using Clampfit 10.7 software to calculate the frequency over 1 minute. The specific operation is as follows: using the Threshold search function in the Clampfit software Event Detection, setting the baseline and threshold for identifying the action potential, and then automatically identifying and measuring each action potential. The curve of firing was plotted using MATLAB. All events, including the spike numbers, were finally verified by eye.

**Determination of mPFC ketamine and 5-HT concentrations**

The animals were euthanized by decapitation, and the mPFC was dissected on an ice-cold plate. For identifying ketamine concentrations after intraperitoneal injection and 5-HT measurements to verify the depletion of 5-HT induced by p-CPA. Ketamine and 5-HT were measured by high-performance liquid chromatography (HPLC). Tissue samples were weighed (30–50 mg of tissue) and homogenized by sonication in 500 μL of homogenization buffer composed of mobile phase [0.1 M citric acid, 0.1 M sodium dihydrogen phosphate, 0.1 mM EDTA, 1.4 mM octane-1-sulphonic acid] and 10 % (v/v) methanol in HPLC grade water spiked with 5 ng/20 μL of N-methyl serotonin (Sigma Aldrich, Ireland). Samples were centrifuged at 15,000×g for 15 min at 4 °C, the supernatants were filtered with syringe microfilters (ultimate AQ-C18, Welch, 2.1× 250 mm, 5 μm), and 10 μL of the filtrate was injected onto a reversed-phase column at a flow rate of 0.8 mL/min for the separation of the ketamine or 5-HT. Concentrations were quantified by electrochemical detection (Antec Decade), and chromatograms were generated using the Class-VP software package (Shimadzu, Japan). The final results were expressed as nanograms of ketamine or 5-HT per gram wet weight of tissue.

**Radioligand binding assay**

The plasmid constructed from human SERT was stably expressed in the human embryonic kidney 293 cells (HEK 293 cells). Then separate the cell membrane SERT from the cytoplasm. The affinities of the test compounds, imipramine (Sigma-I7379) as a positive control, ketamine, and intralipid, for SERT were respectively determined by competitive interaction with [^3^H] Imipramine (WuX, PE-NET576250UC), as previously described. The nonspecific binding compounds (10 uM SB-206553, Sigma-S180; 1 µL) were transferred to the assay plate according to the plate map for nonspecific binding (Low control: LC). DMSO (1 µL) was transferred to the assay plate according to the plate map for total binding (High control: HC). Dispense membrane stocks (100 µL) into the plate by adding radioligand (100 µL). Plates were shaken at 300 rpm under 4 °C for 2 h, and Unifilter 96 GF/C filter plates were soaked with 50 μL of 0.3% PEI per well for 1 h at room temperature. After binding assays, using Perkin Elmer Filtermate Harvester to filter the reaction mixture through GF/C plates, each plate was washed 4 times with cold wash buffer (50 mM Tris-HCl, pH 7.4, 100 mM NaCl). The filter plates were dried for 1 hour at 50 °C. After drying, use Perkin Elmer Unifilter 96 backing seal tape to seal the bottom of the filter plate wells. Next, add 50 μL of Perkin Elmer Microscint 20 cocktail. The top of the filter plates was sealed with Perkin Elmer TopSeal A sealing film. Using the Perkin Elmer MicroBeta2 Reader to count ^3^H trapped on the filter. Calculated the percentage of inhibition using following equation: % Inhibition = (1–(Assay well – Average_LC)/ (Average_HC – Average_LC)) *100%. Analyzing the data with Prism 10 by using the model “Log(inhibitor) vs. response -- Variable slope” to fit the data. Detailed materials and data analysis procedure are in Table S4.

**Serotonin transporter uptake assay**

The plasmid constructed from human SERTs was stably expressed in the human embryonic kidney 293 cells (HEK 293 cells). On the first day, cells were seeded into a 384-well plate at 20000 cells/well in 20 μL. Then incubate cells in the incubator at 37 °C overnight. On the second day, the reference compound [Citalopram (Sigma-PHR1640)] was made in 10 doses of 3-fold serial dilution in assay buffer containing 0.1% BSA at the top concentration of 1 μM using Bravo. The compound (ketamine) was made in 20 doses of 3-fold serial dilution in assay buffer containing 0.1% BSA at the top concentration of 20 mM using Bravo. Transfer 16 μL /well of compound dilutions into the 384 plates by hand and add 24 μL assay buffer to each well. Remove the cell plate from the incubator. Aspirate the medium from the wells by Bravo and transfer 25 μL/well of compound dilutions into the cell plate by Bravo (Note: For High control wells, 25 μL of 0.2% DMSO in assay buffer containing BSA is added. For Low control wells, 25 μL of the top concentration of reference solution is added), 300 rpm, 15 s then incubate at 37 °C for 30 minutes. After incubation of the cells with compounds, add 25 μL of dye solution per well. Incubate at 37 °C for 60 minutes. Finally, read the plate on Envision. Inhibition% = (1 – (Assay well – Low Control average)/ (High Control average – Low Control average)) * 100%. Analyzing the data with Prism 10 by using the model “Log(inhibitor) vs. response -- Variable slope” to fit the data. Detailed assay materials and assay procedure are in Table S5.

**The radiosynthesis and dose formulation of 4-[^18^F]-ADAM**

The precursor for the synthesis of 4-[^18^F]-ADAM was purchased from Huayi Technology. The 4-[^18^F]-ADAM was synthesized as previously reported [101]. Briefly, nucleophilic fluorination of the dinitro precursor with K[^18^F]/K_2.2.2_ (Sigma-Aldrich, St. Louis, MO, USA) followed by reduction with NaBH_4_/Cu(OAc)_2_ (Sigma-Aldrich) and purification with HPLC produced the desired compound with ~5% radiochemical yield (EOB) in a synthesis time of 120 min from the end of bombardment (EOB). The chemical purity of 4-[^18^F]-ADAM was > 95%, and the radiochemical purity was > 98%. The specific molar activity was >3 Ci/μmol. The purified 4-[^18^F]-ADAM was formulated in a solution of 10% physiological saline (0.9%, w/v) in ethanol for intravenous (i.v.) injection into mice.

**PET imaging experiment**

A 90-minute dynamic PET scan was performed using a uMicroExplorer-PET/CT system (Wuhan Lianying Life Science Instrument Co., Ltd.). The mice were divided into two groups, and each group consisted of 6 mice. The control and experimental groups were treated with physiological saline solution (0.9%, w/v) and ketamine (10 mg/kg in Saline), respectively. For the experimental group, the ketamine was administered 5 minutes earlier than the control group. The mice were anesthetized under isoflurane anesthesia (3.0% oxygen) during the PET scans. After tail vein injection of 4-[^18^F]-ADAM, a later 90-minute dynamic PET scan was acquired for each mouse. Each static scan consists of a CT scan and a PET scan. The CT scan time is 60 seconds, and the PET scan time is 90 minutes. The dynamic PET image framing schedule was as follows: 4 × 15 seconds, 4 × 30 seconds, 2 × 60 seconds, 3 × 120 seconds, 2 × 180 seconds, and 2 × 300 seconds. The typical injection volume for each mouse was 100-150 µL (i.v.), and the injection dose was 6-8 MBq.

**PET data analysis**

The PET data were reconstructed with a three-dimensional ordered-subset expectation-maximization (3D-OSEM) algorithm and subsequently analyzed with PMOD software (version 4.3, PMOD Technologies Ltd., Zurich, Switzerland) to align the PET images uniformly to a standard template for accurate localization and segmentation of brain regions. For standardized delineation of the target regions, the animal CT datasets were spatially normalized to the implemented mouse (Ma-Benveniste-Mirrione) CT Atlas. Also, the respective transformation matrices were used to normalize the PET datasets. All transformations were performed using a rigid matching algorithm as implemented in PMOD. Kinetic modeling was performed using the PMOD PKIN module, which employed a two-tissue compartment model (2TCM) to determine the BPnd. The predefined region volumes of interest (VOIs) of the Ma-Benveniste-Mirrione atlas were used to extract mean SUV (standardized uptake value) values from the PET data.

**Microdialysis**

The microdialysis experiments were conducted as in previous research showed with slight modification. Briefly, mice were anesthetized with a mixture of isoflurane (1.0-1.5%) and oxygen (0.6-0.8 L/min), positioned in a stereotaxic instrument. A guide cannula (CMA/7) was unilaterally implanted into mPFC (coordinates: AP, +1.65 mm from the bregma; ML, ±0.40 mm from sagittal structure; DV, –1.60 mm from dura) and fixed with dental cement. After surgery, to acquire a stable baseline for 5-HT, mice were allowed to habituate in a microdialysis apparatus for at least one week. Then, a microdialysis probe (CMA/7, probe length: 1 mm, molecular cut-off: 6 kDa) was implanted into the mPFC through a guide cannula. The flow rate of ACSF (NaCl=142 mM, KCl=3 mM, CaCl_2_=1.2 mM, MgCl_2_=0.8 mM, and Na_2_HPO_4_=3.5 mM, NaH_2_PO_4_=1 mM, PH=7.3) was set as 0.5 μL/min for stabilization. After stabilization for 16 hours, dialysate samples were collected into 4 tubes (15 minutes per tube). At the beginning of the second tube, mice were treated with saline or ketamine (i.p., 10 mg/kg) or memantine (i.p., 10 mg/kg) or AP5 (mPFC, 40 nmol, 300 nL). Following collection, dialysate samples were stored at -80 °C until they were analyzed by LC-MS (ThermoFisher TSQ Altis triple quadrupole mass spectrometer).

**Construct and cell culture**

The full-length human SERT (hSERT, Uniport core: P31645) gene was synthesized and subcloned into pEG BacMam vector[102], with a C-terminal 3C protease cleavage site, an enhanced green fluorescent protein (eGFP), and a StrepII tag. Mammalian HEK293F cells were grown in SMM 293-TII Expression Medium supplemented with 1% FBS at 37 °C and 8% CO_2_. The BacMam virus was generated and amplified in *Spodoptera frugiperda* (SF9) cells cultured in Sf-900 III SFM medium at 27 °C using the Bac-to-Bac baculovirus system. Both cell lines are routinely tested for mycoplasma contamination using CELLshipper Mycoplasma Detection Kit M-100 from Bionique and are mycoplasma-free. No misidentified cell lines were used.

**Expression and purification of hSERT-ketamine**

HEK293F cells with a density of 2.5 x 10^6^ mL^-1^ were infected by the hSERT BacMam virus at an MOI of ~ 2:1. After infection for 12 hours, cells were supplemented with a final concentration of 10 mM sodium butyrate, shifted to 30 °C, and cultured for another 48 hours. The harvested cells were lysed in Lysis buffer containing 20 mM Tris-HCl pH 8.0, 150 mM NaCl, 20 mM n-dodecyl-β-D-maltoside (DDM), 2.5 mM cholesteryl hemisuccinate (CHS), 1 mM phenylmethylsulfonyl fluoride (PMSF), 0.8 μM aprotinin, 2 μg/mL leupeptin and 2 μM pepstatin A for 2 hours at 4 °C. The insoluble material was removed by centrifugation at 50,000 g for 1 hour. The supernatant was filtered through a 0.45 µm filter and incubated with Strep-Tactin resin for 1 hour at 4 °C. The resin was washed extensively with Wash buffer containing 20 mM Tris-HCl, pH 8.0, 100 mM NaCl, 1 mM DDM, and 0.2 mM CHS, and eluted with Wash buffer supplemented with 5 mM desthiobiotin. The eluate was concentrated, incubated with 1:20 (w:w) 3C protease for 1 hour at 4 °C to remove the C-terminal eGFP-Strep tag, and further loaded onto a size-exclusion chromatography (SEC) column (Superose 6 Increase 10/300 GL) pre-equilibrated in a buffer containing 20 mM Tris-HCl pH 8.0, 100 mM NaCl, 0.01% Lauryl Maltose Neopentyl Glycol (LMNG), and 10 µM Ketamine.

**Cryo-EM sample preparation and data collection**

The peak fraction was collected, concentrated to ~4 mg/mL, and incubated with an additional 1 mM Ketamine for 1 hour at 4 °C before freezing grids. 4 µL sample aliquote was applied to a glow-discharged Quantifoil 200-mesh Au 2/1 grid at 8 °C and 95% humidity, blotted for 3 s with a blot force of 0, and flash frozen into the liquid ethane pre-cooled by liquid nitrogen using Vitrobot IV (ThermoFisher Scientific).

Movies were collected on a 300 kV Titan Krios G4 microscope equipped with a Gatan K3 camera and an energy filter, with a dose rate of 20 e-/pix/s. a pixel size of 0.5275 Å (in super-resolution mode), a total dose of 50 e-/Å^2^, and a defocus range from -1.0 to -2.0 µm. The data collection statistics are summarized in Table S2.

**Data processing and model building**

All data processing was performed in cryoSPARC unless otherwise mentioned [103]. 6435 raw movies in super-resolution mode were binned by 2 and motion-corrected with the patch motion correction. Contrast transfer function (CTF) parameters were estimated with patch CTF estimation. 6368 movies with CTF estimation values below 6 Å were retained. Particles were extracted using a box size of 240 pixels with bin-4 and following 2 rounds of 2D classification. Subsequently, three rounds of triplicate *ab initio* were performed, each with increased maximum resolution of 10 Å, 8 Å, and 6 Å, and the initial resolution of 25 Å, 20 Å, and 15 Å, respectively. Good particle stacks in each round were combined, duplicates were removed, and used for the next round. Particles were re-extracted with the bin-1 pixel size for heterogeneous refinement with the *ab initio* model as reference and non-uniform (NU) refinement, improving the resolution to 3.43 Å. The particle stack was further subjected to multiple rounds of local CTF refinement and local refinement, resulting in a 3.27 Å resolution reconstruction.

For model building, an initial model of SERT (PDB 5I6X) was fit in ChimeraX [58, 104] and manually adjusted in COOT [105]. The N-terminal 1-77 residues and C-terminal 618-630 residues could not be built due to the lack of density. Notably, a similar range of residues was also reported from the native porcine SERT structure [58], indicating the flexible nature of the N- and C-termini of SERT. The transmembrane helices were well resolved with clear side chain information (Fig. S2H). A “Y”-shaped density in the central site of SERT, which fit well with ketamine and allowed us to determine its pose and orientation. Lipid-like densities were built with alkanes with different numbers of carbons. The model was refined in real space with Phenix (version 1.20.1-4487).

**Immunohistochemistry and analysis**

To verify the specificity of hM4D(Gi)-mCherry expression in VIP neurons of Vip-Cre mice and to quantify the expression of c-Fos in DRN 5-HT neurons of ePet-Cre mice or different subtypes of mPFC GABAergic neurons (Vip-Cre::Ai47, PV-Cre::Ai47, SST-Cre::Ai47) 1.5 h after intraperitoneal injection of ketamine. All mice were deeply anesthetized with an overdose of pentobarbital and then transcardially perfused with 0.9% saline solution followed by 4% paraformaldehyde (PFA) in PBS. After 24 h of post-fixation in 4% PFA overnight at 4 °C, brains were subsequently immersed in a 30% sucrose solution for dehydration for 24-48 h. Brains were sectioned coronally at a thickness of 40 μm with a Vibration Microtome (Leica VT1200S). Coronal brain sections were washed in PBS, blocking solution containing 0.5% Triton X-100 and 5% normal bovine serum albumin was dissolved in 1x PBS. After blocking for 1-2 h and washing, brain sections were incubated with the primary antibody rabbit polyclonal anti-c-Fos (1:1000, Synaptic system) for 48 h at 4 °C. After washing, these brain sections were incubated with secondary antibody Alexa Fluor 594-conjugated donkey anti-rabbit IgG (1:1000, Jackson ImmunoResearch) at room temperature for 24 h at 4 °C. Cell nuclei were identified using the 4, 6-diamidino-2-phenylindole (DAPI) stain (1:10000, Jackson ImmunoResearch). Imaging was carried out with a digital slide scanner (Olympus, VS120-S6-W) and a confocal microscope (Nikon, Ti-E+A1R SI).

**Supplementary References**

99. Shiue GG, Fang, P, Shiue C-Y. Synthesis of N,N-dimethyl-2-(2-amino-4- [18 F]fluorophenylthio)benzylamine as a serotonin transporter imaging agent. Appl Radiat Isot 2003; 58 : 183–91.

100. Goehring A, Lee CH, Wang KH et al. Screening and large-scale expression of membrane proteins in mammalian cells for structural studies. Nat Protoc 2014; 9 : 2574–85.

101. Punjani A, Rubinstein JL, Fleet DJ et al. cryoSPARC: algorithms for rapid unsupervised cryo-EM structure determination. Nat Methods 2017; 14 : 290–6.

102. Pettersen EF, Goddard TD, Huang CC et al. UCSF ChimeraX: structure visual- ization for researchers, educators, and developers. Protein Sci 2021; 30 : 70–82.

103. Emsley P and Cowtan K. Coot: model-building tools for molecular graphics. Acta Crystallogr D Biol Crystallogr 2004; 60 : 2126–32.
